# An *ex-vivo* Human Intestinal Model to Study *Entamoeba histolytica* Pathogenesis

**DOI:** 10.1371/journal.pntd.0000551

**Published:** 2009-11-17

**Authors:** Devendra Bansal, Patrick Ave, Sophie Kerneis, Pascal Frileux, Olivier Boché, Anne Catherine Baglin, Geneviève Dubost, Anne-Sophie Leguern, Marie-Christine Prevost, Rivka Bracha, David Mirelman, Nancy Guillén, Elisabeth Labruyère

**Affiliations:** 1 Institut Pasteur, Unité de Biologie Cellulaire du Parasitisme, Paris, France; 2 INSERM U786, Paris, France; 3 Institut Pasteur, Unité de Recherche et d'Expertise Histotechnologie et Pathologie, Paris, France; 4 Institut Pasteur, Imagopole, Plate-forme de Microscopie Ultrastructurale, Paris, France; 5 Hôpital Foch, Chirurgie générale et digestive, Suresnes, France; 6 Hôpital Foch, Service d'Anatomopathologie, Suresnes, France; 7 Institut Pasteur, Centre d'analyse médicale, Paris, France; 8 Weizmann Institute, Department of Biological Chemistry, Rehovot, Israel; New York University School of Medicine, United States of America

## Abstract

Amoebiasis (a human intestinal infection affecting 50 million people every year) is caused by the protozoan parasite *Entamoeba histolytica*. To study the molecular mechanisms underlying human colon invasion by *E. histolytica*, we have set up an *ex vivo* human colon model to study the early steps in amoebiasis. Using scanning electron microscopy and histological analyses, we have established that *E. histolytica* caused the removal of the protective mucus coat during the first two hours of incubation, detached the enterocytes, and then penetrated into the lamina propria by following the crypts of Lieberkühn. Significant cell lysis (determined by the release of lactodehydrogenase) and inflammation (marked by the secretion of pro-inflammatory molecules such as interleukin 1 beta, interferon gamma, interleukin 6, interleukin 8 and tumour necrosis factor) were detected after four hours of incubation. *Entamoeba dispar* (a closely related non-pathogenic amoeba that also colonizes the human colon) was unable to invade colonic mucosa, lyse cells or induce an inflammatory response. We also examined the behaviour of trophozoites in which genes coding for known virulent factors (such as amoebapores, the Gal/GalNAc lectin and the cysteine protease 5 (CP-A5), which have major roles in cell death, adhesion (to target cells or mucus) and mucus degradation, respectively) were silenced, together with the corresponding tissue responses. Our data revealed that the signalling via the heavy chain Hgl2 or via the light chain Lgl1 of the Gal/GalNAc lectin is not essential to penetrate the human colonic mucosa. In addition, our study demonstrates that *E. histolytica* silenced for CP-A5 does not penetrate the colonic lamina propria and does not induce the host's pro-inflammatory cytokine secretion.

## Author Summary

*Entamoeba histolytica* is the causative agent of amoebiasis, a human disease. Like other enteric infections, the lack of animal models enhances the difficulty of studying the development of amoebiasis. To date, no experimental model has been developed that reproduces the invasive intestinal amoebic lesions seen in human colon. We present the first study that examines, using human colon explants, the early steps of the human colonic barrier invasion by *E. histolytica*. With this *ex vivo* integrative model we have investigated both parasite behaviour and the human tissue response. Remarkably, in this model *E. histolytica* was able to cross and destroy the intestinal barrier evoking a tissue inflammatory response, while *E. dispar*, a non-pathogenic species, was unable to penetrate nor induce tissue responses. Furthermore, we have explored the role of three virulence factors during the invasive process, using gene-silenced *E. histolytica* trophozoites, particularly the kinetics of invasion, tissue destruction and induction of an early inflammatory responses. This is, to our knowledge, the first time that their role is highlighted in a complex human system. Our study provides new insights in the molecular mechanisms involved in the early steps of human colon invasion by *E. histolytica*.

## Introduction

The protozoan intestinal parasite *Entamoeba histolytica* is the causative agent of human amoebiasis. This disease is primarily a problem in the developing world, where it leads to 50 million clinical cases and 100,000 deaths per year [1]. One of the most puzzling clinical aspects of *E. histolytica* infection is that 90% of individuals are asymptomatic, whereas the remaining 10% develop colitis, diarrhoea, dysentery and (in a few cases) extra-intestinal amoebic lesions, such as liver abscess. The factors that protect the host against invasive diseases and which trigger the invasive process in humans are still poorly understood. However, a link between malnutrition and *E. histolytica* dysentery has been established in infected children in Bangladesh [2]–[4]. Furthermore, genetic reorganization in the parasite [5] and the gender of the host [6]–[8] could play a role in the outcome of the infection. Invasion of the intestinal wall involves several main steps: (i) contact with and degradation of the mucus layer by the trophozoites allowing the amoeba to access the epithelial surface, (ii) intimate adhesion of the amoeba to the mucosal cells enabling expression of its cytolytic activity, and (iii) induction of a host inflammatory response. *E. histolytica* motility is essential for the invasive processes and the demonstrated *in vitro* chemotaxis towards cytokines such as tumour necrosis factor (TNF) and interleukin 8 (IL8) could have a role in directing the migration [9],[10].

A number of animal models for intestinal colitis have been investigated but none reproduces the typical colonic lesions that have been observed in intestinal amoebiasis in humans (for a review, see [11]). The early steps in amoebiasis, such as parasite adhesion to the mucosa, have been investigated in the C3H/HeJ mouse in which chronic infection can be obtained after mechanical injury of the caecal epithelium [12]. Experiments in animal models have shown that once inflammation begins, the epithelial cells release cytokines and chemokines (IL1β, IL8, TNF-alpha, IFN-γ). In the susceptible C3H/HeJ mouse strain, IL-4 secretion was found to regulate the inflammatory response even in the absence of IL-10 and TGF-beta [13]. Furthermore, *E. histolytica* infections were also produced in C57BL/6 IL-10 deficient mice [14]. It has been reported that tissue damage in amoebic colitis in the severe combined immunodeficient mouse-human intestinal xenograft (SCID-HU-INT) arises from both the direct effects of *E. histolytica* on colonic tissue and the resulting gut inflammatory response [15],[16]. Zhang et collaborators have also showed that TNF blockade reduces inflammation and intestinal damage, whereas inhibition of IL-1β reduced cytokine production but had less marked effects on inflammation and disease [16]. Although the inflammatory response produced in the above-mentioned SCID-HU-INT mouse model partially reproduces the early steps of human intestinal parasite invasion, this model lacks lymphocyte responses [17] and it does not take into account the role of the human colonic mucus. Among *E. histolytica* components Gal/GalNAc lectin is a major factor for adherence to mucus [18] and epithelial cells. It has been reported Gal-GalNAc lectin-mediated contact between trophozoites and human epithelial cells and leukocytes induces cell apoptosis [19]. The Gal/GalNAc lectin is a protein complex with a 170 kDa heavy chain and a 35 kDa light chain. The blockage of cell signalling through the lectin in parasites which over-express the carboxyl terminal end of the heavy chain (the substrain HGL-2) significantly reduced amoebic adhesion to epithelial cells and modify abscess formation in animal model [20]. Previous studies have also shown that purified Gal/GalNAc-lectin stimulates the *in vitro* production of TNF by macrophages [16] and induces Toll like receptor-2 expression [21] and dendritic cell activation in Balb/c mice [22],[23], provoking a Th1 immune response. The second factors important for pathogenesis are the amoebapores that kill host cells and are highly homologous to the perforin produced by mammalian NK cells [24],[25]. Investigations in the SCID-HU-INT mouse model with a substrain of *E. histolytica* in which expression of the amoebapore A gene was abrogated, have shown that invasion of the intestinal wall was diminished; however, amoebapore A had no impact on the development of colitis or inflammation [26]. To accomplish pathogenesis amoeba also needs proteases. The *E. histolytica* genome contains at least 40 genes encoding cysteine proteases. Only a few of these are transcribed and even fewer of the proteases are secreted [27],[28]. Nevertheless, studies with an *E. histolytica* substrain in which the expression of several cysteine proteinases was down-regulated by an anti-sense transcript have shown that these proteins play a major role in the development of amoebic colitis in the SCID-HU-INT mouse [29]. One of these cysteine peptidases, EhCP-A5, which is not expressed in *E. dispar* [30] has been shown to degrade the cysteine-rich domains of the MUC2 mucin, the major structural component of the colonic mucus gel in the human digestive tract [31],

Using the colonic explants model [32],[33]. We studied both sides of the host-parasite interaction by determining the kinetics of parasite penetration into the mucus and the mucosa, structural changes in the mucosa, cell lysis and the development of an inflammatory response to virulent wild-type (WT) *E. histolytica* strain (HM1: IMSS) and compared them with those observed for the non-pathogenic parasite *E.dispar*.

We also experimented with a number of substrains of the HM:1:IMSS strain which had been genetically manipulated to produce trophozoites that lacked one or more of the above-mentioned virulence factors. We used HGL-2 trophozoites lacking Gal-lectin activity [20] and strains epigenetically silenced for expression of the amoebapore A gene (AP-A) (strain G3), the light subunit of Gal/GalNAc lectin (Lgl1), (strain RBV) and the cysteine proteinase-5 (EhCP-A5) (strain RB8) [34],[35]. Here, we report that the Gal/GalNAc lectin and amoebapores are not required for invasion of human colon explants and suggest that EhCP-A5 is not required for crossing the mucus but contribute directly or indirectly for penetrating the lamina propria and inducing inflammation.

## Materials And Methods

### Amoeba cultures

The pathogenic *E. histolytica* WT strain used was HM1: IMSS. This virulent strain was also used to produce trophozoites silenced for the *Ehap-a* gene (strain G3) [35] and double-silenced for the *Ehap-a* and *EhLgl1* genes (strain RBV) [34] or *Ehap-a* and *EhCP-A5* (strain RB8) [34]. Amoebae were grown axenically in TYI-S-33 medium at 37°C [36]. The non-pathogenic *E. dispar* (strain SAW 1734) was cultured xenically with *Crithidia fasciculata* in TYI-S-33 at 37°C. [36]. Ttrophozoites transfected with the plasmid pEhExNeo (Neo) [37] or the plasmid containing a truncated *hgl2* gene encoding the transmembrane and cytoplasmic domains of HGL2 (XM_651089.1; HGL2 parasites; [20]) were cultured in the same way, plus supplementation with 10 µg/ml geneticin (G418) (Gibco-BRL). Prior to each experiment, the geneticin concentration was raised to 30 µg/ml for 48 h. All trophozoites were harvested during the logarithmic growth phase (48 h) and collected by centrifugation at 300 g for 5 minutes.

### Human colon explant preparation

Segments of human colon (ascending, descending and sigmoid colon) were obtained from 32 fully informed patients undergoing surgery for colon carcinoma (23 men and 9 women; range age, 47–81 years) and they were analyzed anonymously. Patient written consent was obtained, according to the French bioethics law [38] None of the patients had undergone radiotherapy or chemotherapy. According to the pathologist's examination rules for the longitudinally bisected colon, a healthy segment of tissue which was distant from the tumour region and devoid of metastatic cells was removed. Tissues were processed according to the French Government guidelines for research on human tissues and the French Bioethics Act, with the authorization from the “Institut Pasteur Recherche Biomédicale” investigational review board (RBM./2004.032). The resected tissues were placed in a 50 ml tube containing KREBS medium (117 mM NaCl, 4.7 mM KCl, 1.2 mM MgCl_2_.6H2O, 1.2 mM NaH_2_PO4, 25 mM NaHCO_3_, 2.5 mM CaCl_2_.2H_2_O and 11 mM glucose) at room temperature and transported immediately to our laboratory by an authorized courier. The tissues were dissected under a stereomicroscope in order to remove fat and muscle and to retain the mucus, the mucosa and the submucosa. The explants were cut into segments measuring 3 cm by 1.5–2 cm (i.e. 5.5 to 6 cm^2^) and pinned (with the submucosa facing down) onto a 4% agarose layer in tissue culture Petri dishes (60×20 mm) (Schott Duran, Germany). Trophozoites (8×10^5^ in 1 ml of KREBS medium) from the various amoeba strains mentioned above were added to the luminal face of the colon and incubated in KREBS medium at 37°C for different times (from 1 to 7 hours). Amoeba-free segments served as controls for each experiments and time point. The tissue control (without amoeba) and the tissue incubated with amoeba together with the supernatant of each experiments were analyzed at the same time.

### Cell cytotoxicity assay

Lactate dehydrogenase (LDH) is a well established marker of tissue breakdown and/or cell viability. Thus, for the LDH assay, an aliquot (1 ml) was taken from each explant incubated with 8×10^5^ WT, *E. dispar*, HGL2, NEO, RB8, RBV, G3 or HM1 trophozoites (or in the absence of amoeba) at different time intervals (1 to 7 hours) and was then centrifuged at 2000 g for 5 minutes and stored at −20°C until analysis. The supernatant concentration of LDH was quantified using an enzyme assay (with 1 ml of supernatant) on an automated analyzer, which expressed the results in IU/L (the Dimension® clinical chemistry system from Dade Behring, Schwalbach, Germany, 1971), as described in the manufacturer's instructions.

### Cytokine detection

Cytokine levels were analyzed in the supernatants of explants incubated with 8×10^5^ WT, *E. dispar*, HGL2, NEO, RB8, RBV, G3 or HM1 trophozoites (or in the absence of amoeba) at different time intervals (from 1 to 7 hours) with the Bioplex Protein Array system (Bio-Rad Laboratories) using beads specific for IL-1β, IL-2, IL-4, IL-6, IL-8, IL-10, GM-CSF, IFN-γ, and TNF on the Luminex 100 instrument (Applied Cytometry System, Sheffield, UK), as previously described) [39]. Each sample was tested in duplicate in all experiments.

### Histological analysis

To identify the mucus, Human colonic fragments were incubated without *Entamoeba* and with *E. histolytica* for seven hours, then fixed with Carnoy fixative, stained with Alcian blue and counterstained with haematoxylin. As these conditions were not optimal to preserve and visualise the amoeba after immunostaining, we decided to use an alternative protocol to study the interaction between the amoeba and the mucosa. The tissue architecture was monitored after incubation with (8×10^5^) WT, HGL2, NEO or *E. dispar* trophozoites (or in the absence of amoeba as a control) at 1-hour time intervals up to 7 hours. Eight individual experiments comparing the control tissue, *E. histolytica* wild type and *E. dispar*, as well as three distinct experiments comparing control tissue, *E. histolytica* wild type and the gene silenced trophozoites were performed. Tissues were fixed in 10% formaldehyde in phosphate buffered saline (PBS) for 48 hours and then embedded in paraffin. Three sections (5 µm thickness) were cut from paraffin blocks and stained with standard haematoxylin-eosin (H/E) reagent. The trophozoites were immunostained with a 1∶200 diluted rabbit serum raised against two internal peptides in the heavy chain HGl2 of the Gal/GalNAc lectin (H_2_N-CFNNENKDFIDQYNTN-COOH and H_2_N-CLIKRCNKTSKTTYWE-COOH). For each experiment, a representative image was shown.

### Scanning electron microscopy analysis

The specimens for scanning electron microscopy (SEM) were fixed in 2.5% glutaraldehyde and 2% paraformaldehyde and 0,05% calcium chloride in 0,08 M cacodylate buffer (pH 7,2) overnight at 4°C. Samples were washed three times for 5 min in 0.1 M cacodylate buffer (pH 7.2), post fixed for 1 h in 1% osmium acid and 1,5% potassium iron cyanide in 0.08 M cacodylate buffer (pH 7.2), and then rinsed with distilled water and post fixed in 1% osmium tetraoxide for 1 h at RT. The samples were then rinsed in CaCl_2_ 0,05% in 0.08 M cacodylate buffer (pH 7.2). Samples were dehydrated through a graded series of 25, 50, 75 and 95% acetone solution (10 min each step) and then 100% acetone three times 15 min. After drying in a critical point drier, the specimens were coated with gold palladium and examined under a Hitachi HH-2R microscope.

### Statistical analysis

All LDH and cytokines concentrations are expressed as means and standard deviations (SDs). Inter-group differences (*p* value) were evaluated in Student's unpaired *t*-test using GraphPad software (available online at http://www.graphpad.com). The significant threshold was set to *p*<0.05.

## Results

### Cell lysis in human colonic explants occurs after incubation with pathogenic *Entamoeba histolytica*

**Figure 1:**
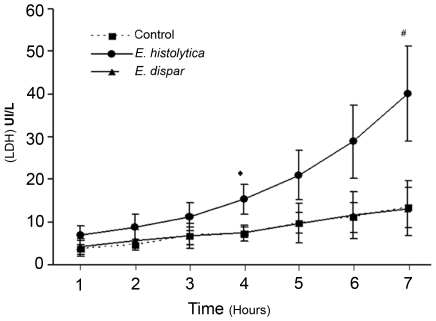
Cell cytotoxicity during interaction between human colonic explants and *E. histolytica* or *E. dispar*. Mean LDH concentrations (IU/L) released into the supernatant of the organotypic culture after incubation with *E. histolytica* WT or *E. dispar* or in the absence of amoeba (control) from 1 to 7 hours. Data are from 8 individual experiments. * indicates a significant difference between WT and control (*p*<0.03) and between WT and *E. dispar* (*p*<0.05). **#** indicates a significant difference between WT and control (*p*<0.001) and between WT and *E. dispar* (*p*<0.02)

In order to determine the impact of pathogenic and non-pathogenic *Entamoeba* on cell viability during organotypic culture of human colonic tissue, we quantified the change over time in lactate dehydrogenase (LDH) enzyme activity in the supernatant. The human colonic segment was incubated (in KREBS medium and at 37°C) with pathogenic *E. histolytica* (the HM1: IMSS strain) or with non-pathogenic *E. dispar* trophozoites for up to seven hours. As shown in Figure 1, the human cells incubated with *E. dispar* released the same levels of LDH as a control tissue in the absence of parasites. In contrast, after four hours of incubation, virulent *E. histolytica* trophozoites lysed human cells and prompted a significant increase in LDH concentration in the supernatant. These results indicate that (i) most of the cells in the human colonic tissue fragment were alive for at least the seven hours of culture and (ii) human cells were killed in the presence of the virulent *E. histolytica* but not in the presence of the non-pathogenic *E. dispar*.

### *E. histolytica* overcomes the protective human colonic mucus layer within two hours

**Figure 2:**
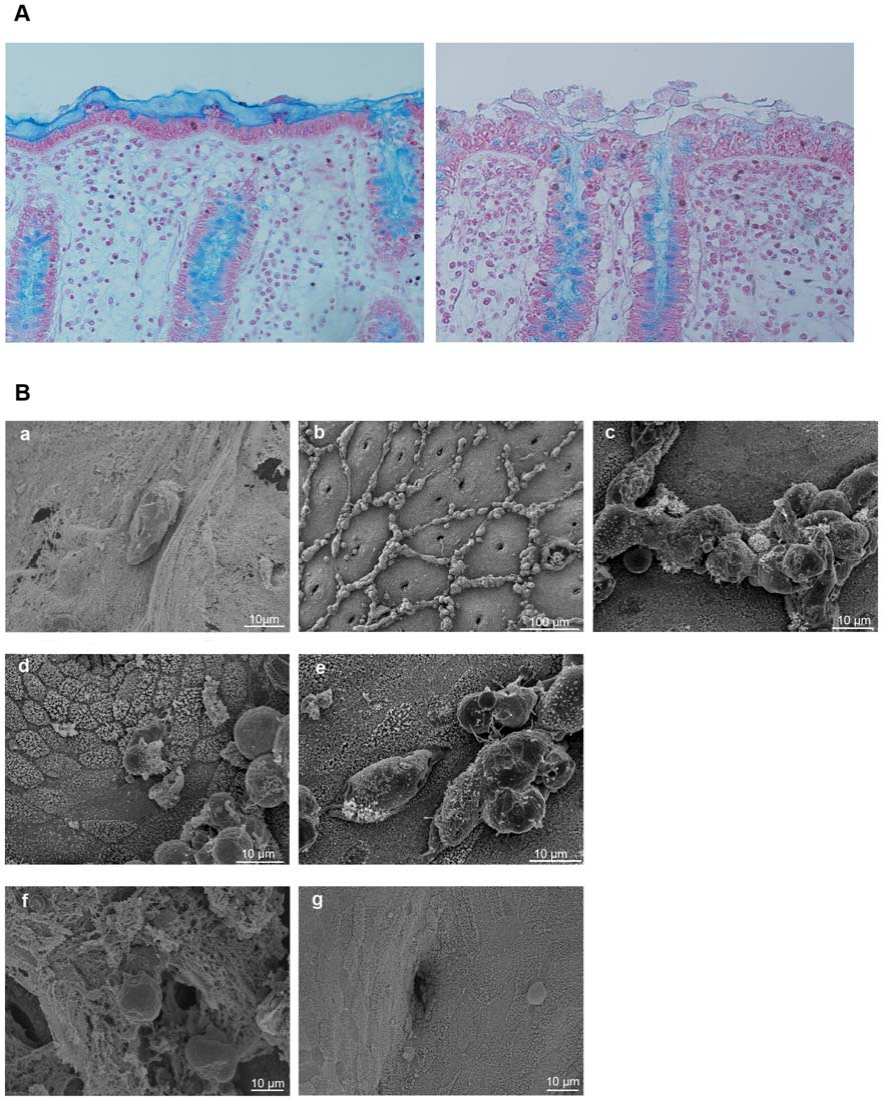
Interaction between Entamoeba and the lumen surface of the human colonic explants. **A**. Analyse by histology of the mucus layer at the surface of Human colonic fragments incubated for seven hours without *Entamoeba* (left panel) and with *E. histolytica* (right panel). The mucus layer covering the epithelium at the surface was observable after seven hours of organotypic culture but not in the presence of *E. histolytica*. **B**. Scanning electron micrographs of the luminal surface of the human colonic explants incubated with *E. histolytica* or *E. dispar*. Representative images from three individual experiments are shown. (a) *E. histolytica* trophozoites adhering to the mucus layer at time 0; (b) 2 hours after incubation, the mucus layer had been degraded by *E. histolytica* and the regular mucosal architecture of the colonic epithelium was visible. Holes corresponded to the crypts of Lieberkühn and abundant aggregates were seen in the interglandular regions. (c) The aggregates were composed of human cells and trophozoites, as seen in an enlargement of this region (d) After 4 hours, the epithelium was damaged and (e) *E. histolytica* trophozoites began to penetrate into the tissue (f) After 4 hours, *E. dispar* trophozoites were still adhering to the mucus but had not degraded it and (g) had not evoked the recruitment of cells to the interglandular region, as shown after manually scraping the mucus after SEM fixation procedure of the sample.

To ensure that a mucus layer protected the human colonic fragments at their luminal surface during organotypic culture, we performed histological analysis using specific staining for mucus (Figure 2.A). After seven hours of organotypic culture, the epithelium was still protected by a mucus layer (left panel). In contrast, after seven hours of incubation, no mucus was found at the surface, suggesting that the trophozoites had caused the removal of the mucus (right panel). In order to visualize the interaction between the pathogenic or non-pathogenic parasites with human colonic tissue, explants incubated with *E. histolytica* or *E. dispar* for two and four hours of incubation were fixed for scanning electron microscopy analysis. In Figure 2.B, the micrographs show a luminal view of the colonic tissue. At the start of the incubation, a thick layer of mucus precludes further observation of the epithelium; nevertheless, *E. histolytica* trophozoites clearly adhered to the mucus, with branching filopodia in contact with the mucus and a tuft of filopodia at the rear (Figure 2. B. a). After two hours of incubation, the mucus layer was no more observable suggesting that it had been removed by *E. histolytica*; the epithelial surface and the crypts of Lieberkühn were visible and an accumulation of material (composed of an agglomeration of human cells and trophozoites) was seen in the inter-glandular region (Figures 2.B. b and c). After four hours of incubation in the presence of the virulent parasite, the epithelium was extensively damaged; the enterocytes were covered by thick microvilli that stuck together (Figure 2.B. d and e). In contrast, after four hours the mucus in the control tissue culture (i.e. in the absence of amoeba, data not shown) or in the presence of *E. dispar*, was still present on the luminal side and the epithelium was not visible (Figure 2.B. f). To find out whether the surface of the epithelium was altered in the presence of *E. dispar*, the mucus layer was mechanically scraped, after the SEM fixation procedure. Accumulated cells were not found and the epithelium had not visibly deteriorated (Figure 2B.g)

### Kinetics of invasion of the colonic tissue and destruction of the mucosal architecture by *E. histolytica*

**Figure 3:**
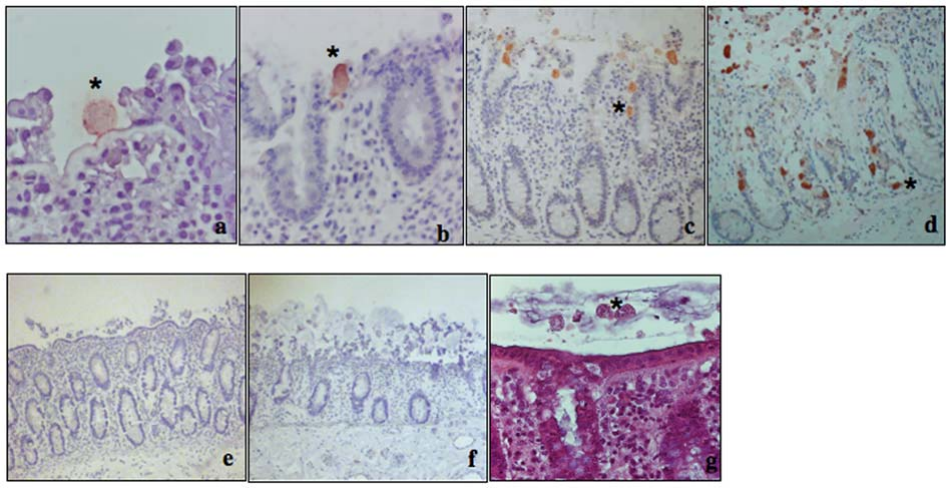
Structure of the mucosa during *E. histolytica*'s invasion of human colonic explants. Time lapse histological studies of the colonic mucosa. Representative images are shown from eight individual experiments; (a) After 2 hours of incubation, *E. histolytica* trophozoites adhered to the epithelium and had detached enterocytes in the interglandular region (b) The trophozoites then migrated along the basal side of the epithelium lining the crypts (c) The trophozoites penetrated deeper into the mucosa after four and (d) seven hours of incubation, leading to the disruption of the mucosal normal architecture. (e) In the absence of amoeba, no alteration of the mucosa architecture was visible, even after 7 hrs of incubation (f) Massive destruction of the mucosal architecture was observed in the presence of *E. histolytica* (g) The non-virulent *E. dispar* was unable to cross the mucus barrier or degrade the epithelium.

Immunohistological analyses of the human colonic explants enabled us to monitor *E. histolytica*'s penetration deeper into the tissue. After two, four and seven hours of incubation with *E. histolytica* and *E. dispar*, human colonic fragments were fixed for histology and longitudinal sections of the tissue were stained or immunostained (using antibodies against the Gal/GalNAc lectin prepared in this work and described in materials and methods section) and analysed. Firm adhesion by *E. histolytica* to the interglandular region and detachment of the enterocytes were observed after two hours of incubation (Figure 3a). Next, the parasites migrated to the crypts of Lieberkühn in order to invade the mucosa (Figure 3b). After four hours of incubation, the epithelial surface was completely degraded (Figure 3c) and the parasites had penetrated deeper into the mucosa. After seven hours, the parasites had left degraded trails behind them (Figure 3d). Surprisingly, the pathogenic trophozoites did not invade the lamina propria indiscriminately but preferred to migrate along the crypts (Figure 3b–d). After seven hours of culture, the mucosa architecture of the control tissue fragment had not been altered (Figure 3e); this contrasted with the architecture of the tissue in contact with *E. histolytica*, which was completely disorganized and, indeed, degraded (Figure 3f). As expected, *E. dispar* was not able to degrade the mucus or penetrate the mucosa. Although these non-pathogenic trophozoites became embedded in the mucus, no alteration of the epithelium was observed (Figure 3g).

### *E. histolytica* induces pro-inflammatory cytokine secretion by the resident cells in human colonic tissue

**Figure 4:**
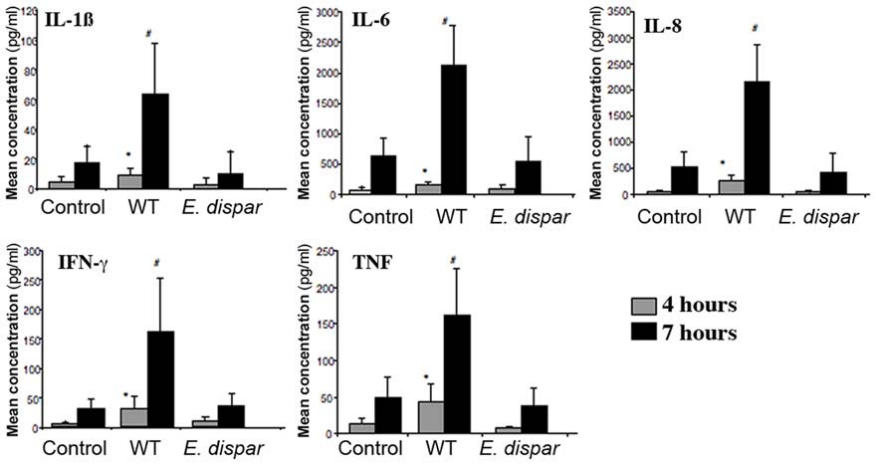
*E. histolytica*-induced secretion of pro-inflammatory cytokines. Histogram showing mean ± SD concentrations (pg/ml) of individual analytes (IL-1β, IL-6, IL-8, IFN-γ and TNF) secreted from 8 human colonic explants incubated with WT, *E. dispar* or without amoeba (control) after 4 and 7 hours of incubation, as measured on a Luminex100 system. Levels of secreted pro-inflammatory cytokines (IL-1β, IL-6, IL-8, IFN-γ and TNF) were significantly higher at 4 hours (* *p*<0.05) and 7 hours (**#** *p*<0.03) in the explants incubated with WT, in comparison with both those secreted by explants incubated with *E. dispar* and the amoeba-free control.

We next sought to establish whether or not segments of human colonic tissue which contains a variety of cell types, including enterocytes, fibroblasts and resident immune cells could develop a specific inflammatory response in the presence of pathogenic *E. histolytica*. To answer this question, we chose to screen and quantify (using multiplexed cytokine bead-based assays) cytokine levels in the supernatant of the tissue incubated with *E. histolytica* and *E. dispar* every hour for seven hours. A broad panel of cytokines has been reported as being present or absent in the various models used to study the inflammatory response during amoebiasis (cell cultures, mice, human intestinal xenografts and patient samples). These include IL-1β, IL-2, IL-4, IL-6, GM-CSF, IL-8, IL-10, IFN-γ and TNF. In our present study, IL-2, IL-4 and IL-10 and GM-CSF were not detected in the supernatant of colonic explants cultured in the presence or absence of parasites (data not shown). After four hours of incubation, the secretion of pro-inflammatory cytokines (such as IL-1β, IL-6, IL-8, IFN-γ and TNF) was significantly higher in the presence of *E. histolytica* but not in the presence of *E. dispar*, compared with the tissue control (Figure 4). Prior to four hours of incubation, no detectable differences in the supernatant concentration of each pro-inflammatory cytokine in each condition were observed. The data obtained after four and seven hours of incubation are presented in Figure 4. The human colonic tissue segments incubated *ex vivo* were rapidly able to develop an inflammatory response in the presence of the pathogenic species *E. histolytica* but not in the presence of the non-pathogenic species *E. dispar*.

### Mucosal cell lysis in the presence of *E. histolytica* impaired in Amoebapore A, Gal-GalNAc lectine or Cysteine Proteinase 5 functions

It has been reported that *Entamoeba histolytica* virulence factors such as the amoebapores, the Gal/GalNAc lectin and the cysteine proteases, play an important role in (i) the killing of mammalian cells, (ii) adhesion to target cells and to mucus and (iii) degradation of mucus and the extracellular matrix (For reviews, [40]–[42]. To further analyze the invasive process of *E. histolytica* within the human colon explants in general and the role of the above-mentioned amoebic factors in particular, we studied *E. histolytica* trophozoites lacking at least one of these components. We used trophozoites with defective Gal/GalNAc lectin signalling (HGL2), trophozoites silenced for the expression of amoebapore A (G3), trophozoites lacking amoebapore A and the light chain (Ehlgl1) of the Gal/GalNAc lectin (RBV) and trophozoites lacking amoebapore A and the cysteine protease CP-A5 (RB8) (for review, [43]).

**Figure 5:**
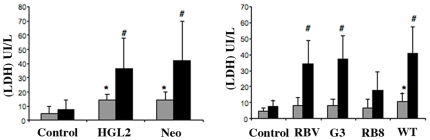
Cell cytotoxicity during interplay between *E. histolytica* trophozoites affected for virulent factors and human colonic explants. Mean LDH concentrations (IU/L, from 3 individual experiments) released after incubation of human colonic explants with HGL2, NEO, RBV, G3, WT and RB8 trophozoites and in the absence of amoeba (control) for 4 hours (grey bars) and 7 hours (black bars). * indicates a significant difference between HGL2 and control (*P*<0.01), NEO and control (*P*<0.03) and WT and control (*P*<0.05). **#** indicates a significant difference between HGL2 and control (*p*<0.008), NEO and control (*p*<0.01), RBV and control (*p*<0.01), G3 and control (*p*<0.009), WT and control (*p*<0.007), RBV and RB8 (*p*<0.03), G3 and RB8 (*p*<0.01) and WT and RB8 (*p*<0.01)

We first examined the cytolytic activity of each the above mentioned strains of trophozoites and compared them with that displayed by trophozoites containing the empty plasmid vector pEhNeo (in the case of HGL2) and with the parental WT strain (for the gene silenced strains). The extent of background cell lysis (which normally occurs during organotypic culture) was determined by the LDH released from enteric cells incubated in the absence of parasites (i.e. a control experiment). The ability of HGL2 trophozoites to lyse human cells was not affected, as shown by the similar quantities of LDH released in the presence of HGL2 and Neo (Figure 5, left panel). pEhNeo trophozoites behaved like WT *E. histolytica* in all our experiments (data not shown). No significant release of LDH was observed after four hours of incubation with G3, RBV and RB8, compared with the tissue incubated in the absence of parasites (Figure 5, right panel). Interestingly, after seven hours of incubation with G3 and RBV trophozoites, the level of released LDH was higher than that of the control tissue and reached the amount obtained with the WT strain, whereas with RB8 trophozoites, the LDH concentration was similar to that of the control tissue. These results indicate that the lack of cell signalling through the Gal/GalNAc lectin and the absence of AP-A did not inhibit *E. histolytica*'s ability to lyse cells. Moreover, in the absence of both AP-A and CP-A5, *E. histolytica* RB8 was incapable of lysing human cells, at least during seven hours of interaction with the colonic tissue.

### Early inflammatory response induced in the presence of HgL2, G3, RBV and RB8 sub-strains of *E. histolytica*

**Figure 6:**
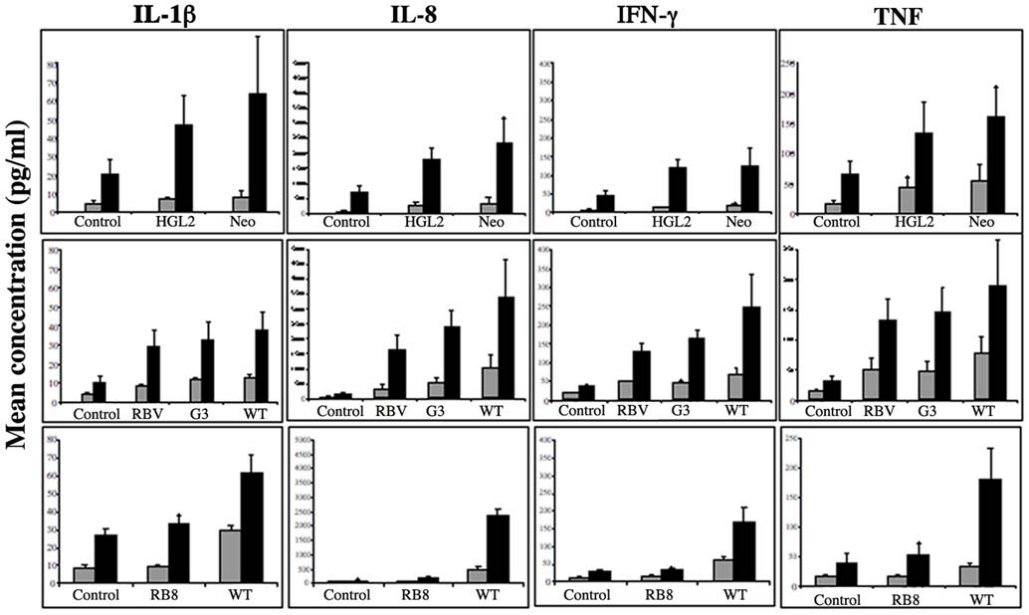
Pro-inflammatory cytokines secretion induced in the *ex-vivo* human colonic model by sub-strains of *E. histolytica*. Mean concentrations (pg/ml) of IL-1β, IL-8, IFN-γ and TNF secreted after 4 hours (grey bars) and 7 hours (black bars) of incubation of HGL2, NEO, RBV, G3, WT and RB8 trophozoites with 3 individual human colonic explants. NEO and HGL2 parasites induced significantly higher levels of pro-inflammatory cytokines IL-1β, IL-6, IL-8, IFN-γ and TNF in the explants incubated for 4 hours (*p*<0.05) and 7 hours (*p*<0.02), compared with the control in the absence of amoeba. RBV, G3 and WT strains induced significantly higher levels of pro-inflammatory cytokines [IL-1β (*p*<0.05,<0.01 and <0.01 respectively) IL-8 (<0.04,<0.01 and <0.01 respectively), IFN-γ(<0.009,<0.003 and <0,01 respectively) and TNF (<0.02,<0.02 and <0.02 respectively)] in the explants incubated for 4 hours and IL-8 (<0.009, <0.001 and <0.01 respectively), IFN-γ (<0.002,<0.0004 and <0.01 respectively) and TNF (<0.008,<0.008 and <0.02 respectively)] at 7 hours, compared with the control in the absence of amoeba. WT secreted IL1β (0.04/0.03), IL-8 (0.001/0.001), IFN-γ (0.001/0.002) and TNF (0.008/0.01) at 4 and 7 hours respectively, compared with RB8.

The human host's inflammatory response to a pathogen is usually triggered by several factors, such as direct stimulation of the immune system by pathogen-associated molecular patterns (PAMPs) and/or factors released by necrotic cells or secreted (i. e. cytokines) by living cells. We thus decided to study the inflammatory reaction of the human colonic tissue in the presence of the strains HGl2, G3, RBV and RB8 by quantifying the supernatant concentrations of the representative pro-inflammatory cytokines IL1β, IL8, IFNγ and TNF after four and seven hours of incubation. In the presence of HGL2 trophozoites, the supernatant concentrations of the four tested cytokines were significantly higher at four and seven hours than those obtained with the tissue alone but were similar to those obtained in the presence of the control (Neo) trophozoites (Figure 6, upper panel). It is important to note that the HGL2 parasites still display the heavy chain of the Gal/GalNAc lectin at their cell surface. Nevertheless, our data indicated that inhibition of signalling through the Gal/GalNAc lectin to the actin cytoskeleton did not affect the tissue inflammatory process. The concentrations of IL1β, IL8, IFNγ and TNF in the supernatant of the human colonic fragment incubated with either the silenced strain G3 or RBV did not differ significantly from those obtained in the presence of the WT strain at each time point (i.e. four and seven hours of incubation) (Figure 6, middle panel). In contrast, there was a significant difference between the supernatant cytokine levels of colonic fragments incubated with the WT and those from the silenced parasite RB8. The concentrations of IL1β, IL8, IFNγ and TNF after incubation with RB8 did not differ from those obtained in organotypic culture, as shown in Figure 6 (lower panel).

### Capacity of *E. histolytica* sub-strains to invade the lamina propria in human colonic explants

**Figure 7:**
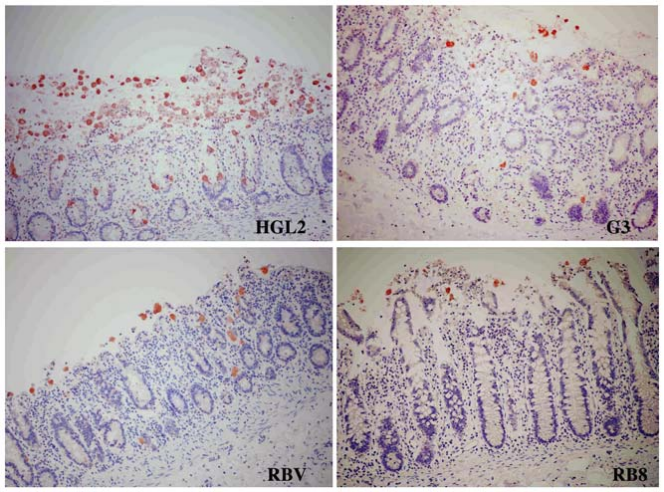
Migration through the lamina propria of *E. histolytica* sub-strains impaired in virulent functions. Representative images from three individual experiments are shown. Histological examination of colonic tissue sections after seven hours of incubation, with HGL2, G3, RBV and RB8. Transversal tissue slices were stained with haematoxylin-eosin. Trophozoites were immunostained with antibodies against the Gal/GalNAc lectin. Experiments with HGL2, G3 and RBV revealed that trophozoites were able to invade the mucosa, as described for the WT. In contrast, RB8 parasites were unable to penetrate deeper into the lamina propria and were blocked at the surface of the mucosa, although they were still able to disorganize and detach cells from the upper side of the mucosa.

In order to establish whether the trophozoites HGL2, G3, RBV and RB8 were capable of invading the human colonic barrier, we examined the tissue ultrastructure and ascertained the presence of the trophozoites. After two, four and seven hours of incubation with either HGL2, pEhNeo, G3, RBV, RB8 or WT trophozoites, the tissues were fixed and processed as described for histological analysis and immunostaining (with anti-Gal/GalNAc lectin antibodies). After two and four hours of incubation, there were no major differences between the strains regarding the degradation of the epithelial surface (data not shown). After seven hours of incubation, HGL2, G3 and RBV trophozoites had penetrated into the lamina propria, as had the pEhNeo and WT trophozoites (Figure 7). Strikingly, the RB8 trophozoites were unable to penetrate the mucosa, although they were capable of reaching its surface and disorganizing its architecture (Figure 7).

## Discussion

Like other enteric infections, one of the main drawbacks of experimental amoebiasis is the lack of an adequate animal model that is capable of reproducing the invasive lesions which occur in the large intestine. As humans are the only hosts known to develop amoebiasis, we decided to use human colon explants as an *ex vivo* model for experimental analysis of the major pathogenic factors leading to this infectious disease. The *ex vivo* human colonic model enables us to study the early stages of infection, the integrity of the colonic tissue during the invasive process and the host immune response. Advantageously, using human colon explants means that there is no need to extrapolate from rodent models to humans and that all experiments are performed with an integrated model combining human cells and molecules present in the colonic mucus and the enteric mucosa and submucosa. In all, we used 32 human colon samples in the present experiments originating from patients of different gender or age and isolated from different parts of the colon, but we did not find any major change in the tissue responses during our experiments. By using a cytotoxicity assay, we established that no significant lysis of human cells occurred during at least seven hours of organotypic culture or in the presence of the non-pathogenic *E. dispar*. In contrast, a significant level of cell lysis was observed after four hours of interaction with a pathogenic strain of *E. histolytica*, indicating that the human cytolysis was specifically due to the presence of virulent parasites.

The time course of *E. histolytica*'s invasion of human colon explants was established using SEM and histological analysis. We observed that *E. histolytica* trophozoites caused the removal of the mucus and reached the epithelial surface within two hours, after which time the parasites invaded the mucosa, detached the enterocytes and migrated along the crypts of Lieberkühn. The specific attachment of trophozoites to the interglandular region of the human colonic epithelium (and not the luminal surface) has also been described in a rodent closed caecal loop model [44]. Shedding of apoptotic cells in the interglandular region suggests that this site is preferentially targeted by *E. histolytica* and is a fragile region that could facilitate penetration of the virulent parasite or the phagocytosis of apoptotic cells. Furthermore, the fact that the trophozoites did not cross and migrate everywhere in the lamina propria but followed the trail formed by the epithelium suggests that in order to migrate, the parasites should require a signal present in the epithelial basement membrane's dense extracellular matrix.

The inflammatory response and cytokine release following host cell-parasite interaction have been previously demonstrated *in vitro* with cultured cell monolayers [45],[46], *in vivo* in several animal models [17],[26],[29] and most recently in patients infected with *E. histolytica* and *E. dispar* [47]. There is increasing evidence that the parasite's presence evokes an immune response (characterized by the secretion of pro-inflammatory mediators) by the intestinal epithelial cells and in which the latter act as antigen-presenting cells [15],[45]. Histological analysis of human colonic biopsies has revealed slight infiltration of neutrophils, macrophages and dendritic cells into the submucosa at the start of the ulceration process. An increase in the neutrophil, plasma cell, eosinophil, macrophage and T cell counts is observed as the infection progresses [48]. In the present study, we used a histological technique to observe the presence of resident immune cells (such as monocytes and T-lymphocytes which are capable of contributing to the tissue inflammatory response during amoebiasis) within the lamina propria of the colonic explants. In order to analyze the relevance of the explant's inflammatory response, we measured the supernatant concentration of a panel of cytokines. We found that pro-inflammatory cytokines (IL-1β, IL-6, IL-8, IFN-γ and TNF) were significantly and specifically secreted in the presence of *E. histolytica* but not in the presence of *E. dispar*. These results are in agreement with previous findings in the SCID-HU-INT model, in which IL-1β and IL8 are produced in response to *E. histolytica* trophozoites introduced into the engrafted human intestinal segments [17]. The release of pro-inflammatory cytokines by the epithelium appears to be an effective means of initiating a mucosal inflammatory response. Other cytokines (such as IL-2, IL-4 and IL-10) were not detected in the supernatant of the colonic explants cultured in the presence or absence of parasites. These findings are also in agreement with the earlier reports showing low IL-10 production during *E. histolytica* infection in both susceptible mice and a human epithelial cell line [2],[49]. Moreover, Hamano et al (2006) [14] successfully produced *E. histolytica* infections in C57BL/6 IL-10 deficient mice. The high levels of pro-inflammatory cytokines induced here by *E. histolytica* trophozoites demonstrated that the *ex vivo* human model can be exploited to study the initiation of inflammatory responses at early stages in amoebiasis and that the explants' responses were specifically induced by amoebic virulence factors.

In the present study, we investigated the role of key virulence factors such as the Gal/GalNAc lectin, amoebapore A and CP-A5,in the invasive process and inflammatory responses in the *ex vivo* human model. We analysed the interaction between the human colon explants and four *E. histolytica* sub-strains: a strain affected in the Gal/GalNAc lectin signalling HGL2, [20], strains silenced for the expression of amoebapore A (G3) [35], the amoebapore A and the Gal/Gal/NAc lectin light subunit 1 (RBV) [34] and the amoebapore A and CP-A5 (RB-8) [34]. The strains with impaired Gal/GalNAc lectin (whether through the heavy chain (HGL2) or the light chain 1 (RBV)) were not inhibited with respect to the invasive process, suggesting that other molecules are required to adhere to the epithelium and then to transduce signals to the cytoskeleton for migration during intestinal amoebiasis.

Although the Gal/GalNAc lectin has been described as a key component in binding mucus and target cells, other molecules like EhADH 112 (which binds red blood cells [50],[51] and KERP1 (which adheres to enterocytes) [52] could have a role in trophozoite adhesion to and then migration through the colonic barrier. These results emphasize the point that the generation of intestinal amoebiasis or hepatic amoebiasis are not induced by the same virulence factors, as neither HGL2 nor RBV trophozoites were able to develop large liver abscesses in the hamster model [20],[34],[53] but were still capable of intestinal invasion.

This is also true of amoebapores, since an amoebapore A-deficient strain was incapable of inducing liver abscess formation in a severe combined immunodeficiency (SCID) mouse model but was still able to cause inflammation and tissue damage in human colonic xenografts patched in the same model [26]. The results obtained in the present *ex vivo* model also demonstrate that the amoebapore-deficient G3 strain was not inhibited in terms of the invasive process or induction of an inflammatory response. For *in vitro* growing conditions, the amoebapore silenced strain, G3, has been found to have numerous off-target silenced genes in comparison to the parent strain HM-1 (I. Bruchhaus and D. Mirelman, unpublished data). Nevertheless, our results show that the G3 strain as well as the RBV strain had a similar behaviour as the parent HM-1 strain in their ability to invade the colonic mucosal surface and to trigger an inflammatory response.

Cysteine proteases have been shown to have an essential role in both hepatic and intestinal amoebiasis. Trophozoites in which only 10% of cysteine protease activity was retained fail to induce liver abscess and intestinal epithelial cell inflammation [29],[54]. These trophozoites were also ineffective at crossing the protective mucus layer produced by cell lines in culture [55]. Recently, Bracha *et al.* [34], have demonstrated the role of CP-A5 in the development of liver abscess using a strain silenced for expression of this protease. Interestingly, RB8 strain was able to cross the mucus barrier in our *ex vivo* colonic model but was unable to migrate within the mucosa or evoke an inflammatory response during the seven hours of incubation. The behaviour of the trophozoite lacking CP-A5 suggested that (i) other proteases than CP-A5, which are produced by *E. histolytica* RB8 appear to be involved in the removal of the colon mucin gel; (ii) CP-A5 may have an essential role in degradation of the extracellular matrix. In agreement with this hypothesis, it has been shown that CP-A5 possesses collagenase activity [56]. Furthermore, it has been shown *in vitro* that *E. histolytica* is attracted by TNF and IL8 [10],[57]; one can hypothesise that in the absence of inflammation, trophozoite migration was not directed towards the human tissue. RB8 trophozoites were not able to induce human cells lysis, as demonstrated by the absence of LDH release. However, when examining the histological sections, we observed detached human cells - suggesting that cells were nevertheless dying and that the lack of an inflammatory response could be linked to the lack of cell lysis by the RB8 strain lacking CP-A5. In a transcriptomic comparison for proteinases gene expression between G3 and RB8 strains growing *in vitro*, the only gene that was silenced in the strain RB8, was the *CP-A5* gene (I. Bruchhaus and D. Mirelman, unpublished data). This has been also shown at the protein level, using a radiolabelled inhibitor of cysteine proteases, that covalently bound to all the cysteine proteases of *E. histolytica* and it revealed that only CP-A5 was not present in RB8 [34]. However, at this point we do not have information on gene expression of G3 or RB8 strains in an *in vivo* invasive situation in which a more complex regulation of gene expression can occurs suggesting that the absence of CP-A5 and/or the expression of non yet identified genes could account for the phenotype obtained for the CP-A5 silenced trophozoites in contact with human colon fragments. Further studies are in progress, to analyze additional roles of CP-A5 as well as the possible impact of other amoebic molecules and the effects of colonic components (such as the bacterial flora) on tissue invasion parameters.

In conclusion, we have shown that the human colonic explant is an integrated, improved model that enables assessment of the interplay between *E. histolytica* and colonic tissue at the early step of infection. This model will help us better understand intestinal amoebiasis in general and the molecular mechanism of invasion by *E. histolytica* and the inflammatory responses evoked by the human tissue in particular. By using this model, we expect to identify key molecules in the host-pathogen interaction and which may have a role in the switch between commensal and virulent forms of *E. histolytica*.
